# A case of eosinophilic granulomatosis with polyangiitis combined with pulmonary tuberculosis: A case report

**DOI:** 10.1097/MD.0000000000039721

**Published:** 2024-09-13

**Authors:** Yuting Lai, Shan Xiao, Yan Shen

**Affiliations:** a Department of Respiratory, Longgang Central Hospital of Shenzhen, Shenzhen, Peoples’s Republic of China.

**Keywords:** eosinophilic granulomatosis with polyangiitis, glucocorticoids, pulmonary tuberculosis, treatment

## Abstract

**Rationale:**

Eosinophilic granulomatosis with polyangiitis (EGPA) is a rare autoimmune disease that can affect multiple organ systems. The standard treatment mainly relies on glucocorticoids and immunosuppressive agents. In our study, we present an EGPA patient who had pulmonary tuberculous mycobacteria infection, such cases are rarely reported.

**Patient concerns:**

A 71-year-old male patient was diagnosed with EGPA (systemic type) and pulmonary tuberculosis simultaneously.

**Diagnoses:**

The Five-Factor score indicated that the patient required glucocorticoids combined with immunosuppressive agents for induction therapy, however, the use of immunosuppressive agents would significantly inhibit antituberculosis treatment. Nowadays, treating active autoimmune disease in patients with infections remains a clinical challenge.

**Interventions:**

Considering the patient did not show life-threatening or severe organ involvement and reduced the effect of antituberculosis immunity, we used glucocorticoids alone.

**Outcomes:**

Finally, the patient had no adverse events, the eosinophil counts were markedly decreased and symptoms of EGPA were relieved.

**Lessons:**

The patient of EGPA combined with pulmonary tuberculosis successfully treated with glucocorticoids alone may provide significant support in selecting the appropriate treatments for similar cases in the future.

## 1. Introduction

Eosinophilic granulomatosis with polyangiitis (EGPA) is associated with antineutrophil cytoplasmic antibody-associated vasculitis.^[1]^ Although EGPA is a type of antibody-associated vasculitis, only 30%–40% of EGPA patients test positive for antineutrophil cytoplasmic antibodies (ANCA). Based on the type of target antigen, ANCA can be divided into myeloperoxidase (MPO)-specific ANCA (MPO-ANCA) and proteinase 3 (PR3)-specific ANCA (PR3-ANCA). Compared to ANCA-negative EGPA patients, those with ANCA-associated EGPA exhibit more characteristics of vasculitis.^[2]^ There is still a lack of understanding of the pathogenesis, pathological alterations, and clinical characteristics of EGPA. It has been reported that the condition of EGPA patients can rapidly deteriorate and lead to death within 6 months.^[3]^ Therefore, accurate diagnosis and timely treatment are extremely crucial.

The treatment of EGPA remains a controversial topic. The symptoms of EGPA can be alleviated by glucocorticoids alone sometimes. However, for systemic EGPA, glucocorticoids and immunosuppressive agents are often used in combination. Additionally, extra therapy is required for EGAP that is recurrent or resistant to treatment.^[4]^ Generally, a Five-Factor score is used to evaluate the presence of poor prognostic before making a treatment decision. The Five-Factor score was originally developed by the French Vasculitis Study Group and was revised by Pagnoux et al in 2007 and is widely used for prognosis evaluation.^[5]^ Glucocorticoids are the basis of EGPA treatment and can significantly improve the remission rate and lower disease-related mortality.^[6]^ According to some research, glucocorticoids by themselves can also have positive results in EGPA.^[7]^

Pulmonary tuberculosis is a common chronic infectious disease caused by *Mycobacterium tuberculosis* infection.^[8]^ Studies have found that patients with systemic necrotizing vasculitides have a higher risk of developing tuberculosis in the first stage of their disease. Additionally, granulomatosis with polyangiitis (GPA) and polyarteritis nodosa are independent factors related to tuberculosis.^[9]^ It is unknown if EGPA patients have a greater incidence of tuberculosis than the general population. According to Yang et al,^[10]^ a case of EGPA was misdiagnosed for 3 years as asthma and pulmonary tuberculosis, and was eventually identified as having EGPA through a skin biopsy. After receiving glucocorticoid therapy the symptoms subsided.^[10]^ The research mentioned above indicates some clinical similarities between EGPA and asthma/tuberculosis, but fundamentally distinct therapeutic approaches. Therefore, in order to prevent improper treatment, a thorough and methodical clinical evaluation is required for prompt and accurate discrimination.

## 2. Case presentations

A 71-year-old male patient was admitted to the respiratory department of the Longgang Central Hospital (Shenzhen, China) with a complaint of “repeated wheezing for 2 years, and recurrence accompanied by chest pain for 10 days.”

Blood routine tests showed an elevated eosinophil ratio of 15.2% and an absolute basophil count of 0.96 × 10^9^/L. Moreover, serological tests showed that IgE of 2447 IU/mL, ANA of 681 AU/mL, and dsDNA of 2.55 IU/mL were significantly increased. Furthermore, we detected ANCA in the serum through immunoassay and the results showed that the anti-MPO, anti-PR3, and anti-GBM tests were negative. The other laboratory test results are shown in Table 1.

**Table 1:** The laboratory tests.

Variables	Results
Complete blood cell count	
White blood cell count (10^9^/L)	6.31
Lymphocyte (%)	18.9
Monocyte (%)	7.8
Eosinophil (%)	15.2↑
Absolute eosinophil count (10^9^/L)	0.96↑
Red blood cell (10^9^/L)	4.59
Hemoglobin (g/L)	141
Platelet count (10^9^/L)	214
Liver and renal function, HCRP	
Total bilirubin (μmol/L)	12.8
Total biliary acid (μmol/L)	1.7
Albumin (g/L)	42.3
Globulin (g/L)	31.2
ALT (U/L)	12
AST (U/L)	13
Creatinine (μmol/L)	63.6
High sensitivity C reaction protein (mg/L)	<0.5
Serologic testing	
Immunoglobulin E (IU/mL)	2447↑
dsDNA (IU/mL)	2.55↑
Antinuclear antibodies (AU/mL)	681↑
Anti-MPO	Negative
Anti-PR3	Negative
Anti-GBM	Negative

ALT = alanine aminotransferase, AST = aspartate aminotransferase, dsDNA = anti-dsDNA antibody, GBM = glomerular basement membrane, MPO = myeloperoxidase, PR3 = proteinase 3.

**Figure 1.**
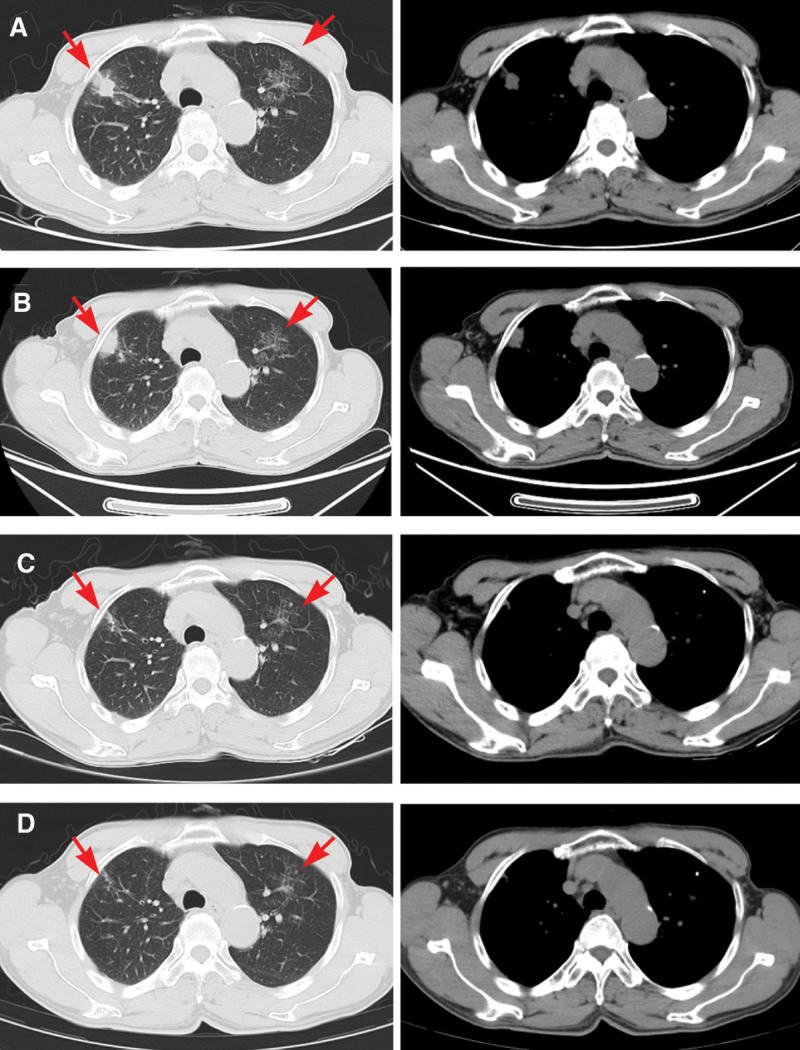
Computed tomography images of the patient. (A) Before 1 d in hospital, CT images showed irregular nodule in the left upper lobe and patchy opacity in the left upper lobe of the lung. (B) After 10 d in hospital, CT images at the initial diagnosis showed also irregular nodule in the left upper lobe and patchy opacity in the left upper lobe of the lung. (C) After received treatment for 6 wk, CT images showed a reduction in size of irregular nodule and decreased in extent of patchy opacity. (D) After received treatment for 9 mo, CT images showed a significant reduction in size of irregular nodule and with residual cord-like opacities remaining. CT = computed tomography.

**Figure 2.**
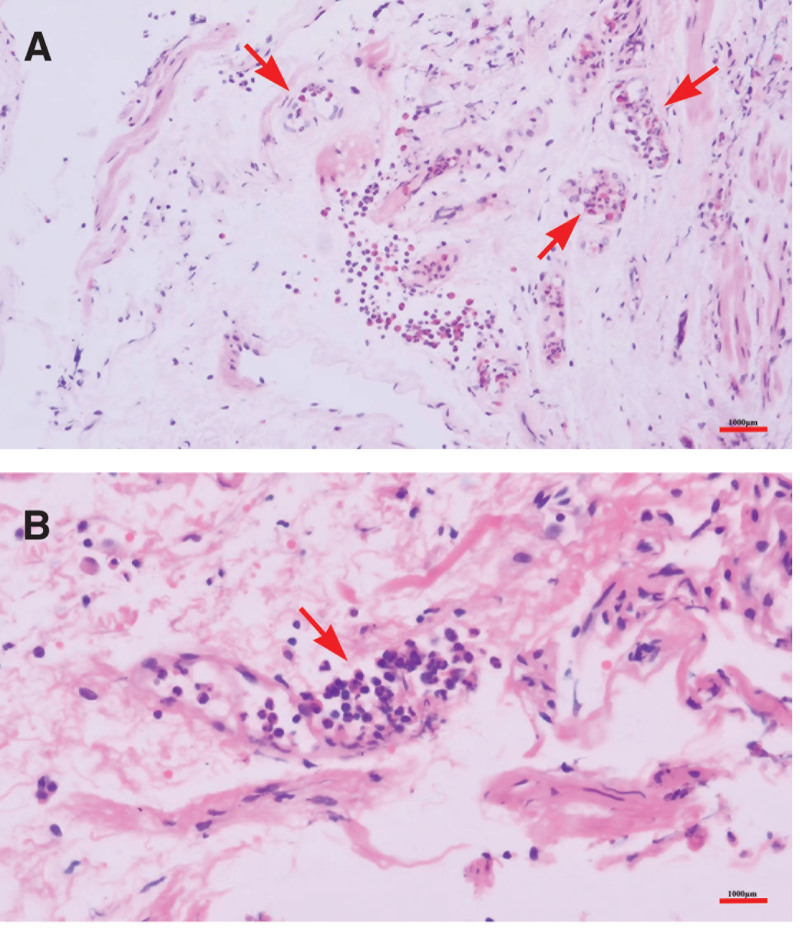
Histopathological examination with hematoxylin and eosin showing the vessels of lung tissue from the right upper lobe apex (A), eosinophilic infiltration of lung tissue from the right upper lobe apex (B). The length of the scale bar as 1000 μm.

Chest computed tomography (CT) scan showed patchy, nodular, and strip-like high-density shadows in the left upper lung, and, there were irregular nodules and patchy consolidated shadows with irregular shapes in the upper anterior segment of the right upper lung (Fig. 1A and B). A minor quantity of local bronchial slight dilation and inflammation was indicative of tuberculosis. Multiple eosinophils were discovered by histopathology in the localized interstitial, vascular cavity, and vascular wall of the lung tissue, and no vasculitis with eosinophilic infiltration has been found (Fig. 2A and B).

Pulmonary function tests showed FEV1/FVC of 68.46%, which is lower than the normal reference value of 70%. This usually indicates the presence of obstructive airway disease. The absolute of FEV1 is 2.97, after inhaling acetylcholine, the patient’s FEV1 decrease to 2.21, with a decrease of more than 20%. The positive challenge test indicates the presence of airway hyperreactivity. Meanwhile, the findings of the Bronchial Provocation Tests revealed a considerable increase in FeNO to 70 ppb. Furthermore, nerve conduction studies showed many peripheral sensory nerve injuries.

Microbiological tests including acid-fast staining and sputum culture, both of them were negative. However, X-pert yielded positive results from bronchoalveolar lavage fluid with *M tuberculosis*. In addition, the X-pert results from the bronchoalveolar lavage fluid showed that rifampin resistance was negative.

Given the asthmatic symptoms, the peripheral eosinophil ratio was elevated at 15.2%, multiple peripheral neuropathy and extravascular eosinophils, the diagnosis of EGPA was made according to the 2022 American College of Rheumatology criteria.^[11]^ In our case, the patient is older than 65 years, furthermore, the patient has no otorhinolaryngological symptoms, and a paranasal sinus CT scan was negative, there was no evidence of involvement in the ear, nose, and throat regions. Taking into account the above 2 points, the total score is 2. This suggests that the patient may have a poorer prognosis.

For systemic EGPA, glucocorticoids and immunosuppressive agents are frequently combined. However, due to the infection of *M tuberculosis*, the patient received oral prednisone alone without immunosuppressive agents in order to protect EGPA’s antituberculosis immunity. We initially administered prednisone acetate at a dosage of 50 mg once daily, which was then tapered to 45 mg once daily after 3 months, 40 mg once daily after 4 months, 30 mg once daily after 5 months, 10 mg once daily after 7 months, and finally maintained at 5 mg once daily after 10 months. Additionally, antituberculosis drugs (isoniazid + rifampin for 10 months), and amebicide (etambutol + pyrazinamide for 2 months) treatment were also implemented.

During the 10-month follow-up after discharge, the patient reported no any adverse events of glucocorticoids and antituberculosis drugs, chest pain, and wheezing symptoms were relieved. Meanwhile, the patient’s chest pain and sensory disturbances in both upper limbs disappeared. Most importantly, the counts of eosinophil returned to normal. Reexamined chest CT showed significant absorption in the right upper lobe and left upper lung (Fig. 1C and D).

## 3. Discussion

EGPA is a small-vessel vasculitis associated with ANCA, characterized by eosinophilia infiltration.^[1]^ EGPA can involve multiple systems and organs of the body, with complex and diverse clinical symptoms. In clinical practice, misdiagnosis and missed diagnosis can readily occur for a variety of reasons. The patient in our study has a history of asthma, the eosinophilic counts were markedly elevated in peripheral blood, and the lung tissue biopsy showed significant eosinophilic infiltration. Moreover, this patient suffered damage to their sensory nervous system, one of the early signs of EGPA. In addition, studies have shown that only 35.8% of EGPA patients are ANCA-positive,^[12]^ in our patient, there was no ANCA antibody was detected. Considering the aforementioned, an EGPA diagnosis was made.

Accumulating studies reported that the risk of tuberculosis is increasing in our country. If *M tuberculosis* is not detected due to long-term hormone therapy, it may result in the spread of tuberculosis in EGPA. At present, there are several methods have been applied to detect tuberculosis, including culture, smear, X-pert, etc. Culture may take a long time and has a low positive rate.^[13]^ The X-pert method has obvious advantages in detecting *M tuberculosis* in patients with suspected pulmonary tuberculosis.^[14]^ In this case, *M tuberculosis* in the bronchoalveolar lavage fluid was identified by X-pert, although acid-fast staining and culture of the patient’s sputum were negative. Therefore, we recommended that patients with suspected pulmonary tuberculosis undergo X-pert testing to improve the diagnostic positive rates. Combined with the patient’s symptoms, imaging characteristics, and pathogen detection results, the diagnosis of pulmonary secondary tuberculosis is confirmed. As a result, we consider that the diagnosis of EGPA (systemic type) combined with pulmonary tuberculosis is established.

EGPA patients combined with tuberculosis infection are rarely reported, and treatment remains challenging. On the 1 hand, high-dose or prolonged use of immunosuppressive agents can lead to a decrease of lymphocytes, monocytes, macrophages, neutrophils, etc, which makes the body more susceptible to various infections, especially *M tuberculosis*.^[15]^ On the other hand, studies reports suggest that the development of ANCA is associated with *M tuberculosis* infection. One case report found that a patient with non-tuberculosis mycobacterium infection developed ANCA-associated vasculitis after 1 year.^[16]^ The studies indicated that non-tuberculosis mycobacterium infection may induce ANCA. At the same time, it has also been reported that the use of antituberculosis drugs such as rifampicin and isoniazid may induce ANCA-associated vasculitis^[17]^ and ANCA were present in 44.4% of tuberculosis patients by indirect immunofluorescence.^[18]^

## 4. Conclusion

In conclusion, we successfully treated the patient of EGPA (systemic type) complicated by pulmonary tuberculosis. The patient’s Five-Factor score was 2 points. Therefore, glucocorticoids and immunosuppressants should be used in combination for treatment. However, the use of immunosuppressants may cause the spread and recurrence of tuberculosis. With both of these advantages and disadvantages, we treated with only glucocorticoids on the basis of antituberculosis therapy, and the condition of the patients markedly improved. Our case can provide important clues for physicians in selecting appropriate treatment in patients with EGPA complicated by pulmonary tuberculosis.

## Acknowledgments

The authors thank all the staff for their valuable contribution to the study.

## Author contributions

**Formal analysis:** Yuting Lai.

**Investigation:** Yuting Lai, Shan Xiao.

**Resources:** Yuting Lai.

**Writing—original draft:** Yuting Lai, Shan Xiao.

**Writing—review & editing:** Yuting Lai.

**Data curation:** Shan Xiao.

**Methodology:** Shan Xiao.

**Software:** Shan Xiao.

**Visualization:** Shan Xiao.

**Conceptualization:** Yan Shen.

**Project administration:** Yan Shen.

**Supervision:** Yan Shen.

**Validation:** Yan Shen.
